# Supplementary material for: Assigning a role for chemosensory signal transduction in Campylobacter jejuni biofilms using a combined omics approach
Source: Sci Rep. 2020 Apr 22;10:6829. doi: 10.1038/s41598-020-63569-5 (PMC7176700; doi:10.1038/s41598-020-63569-5)
Supplement: Supplementary file 1 — Supplementary information. [file 41598_2020_63569_MOESM1_ESM.pdf]

Assigning a role for chemosensory signal transduction in *Campylobacter jejuni* biofilms using a combined omics approach

Greg Tram[1¶], William P. Klare[2,3¶], Joel A. Cain[2], Basem Mourad[1], Stuart J. Cordwell[2,3], Victoria Korolik[1*&], Christopher J. Day[1*&]

Supplementary Figures

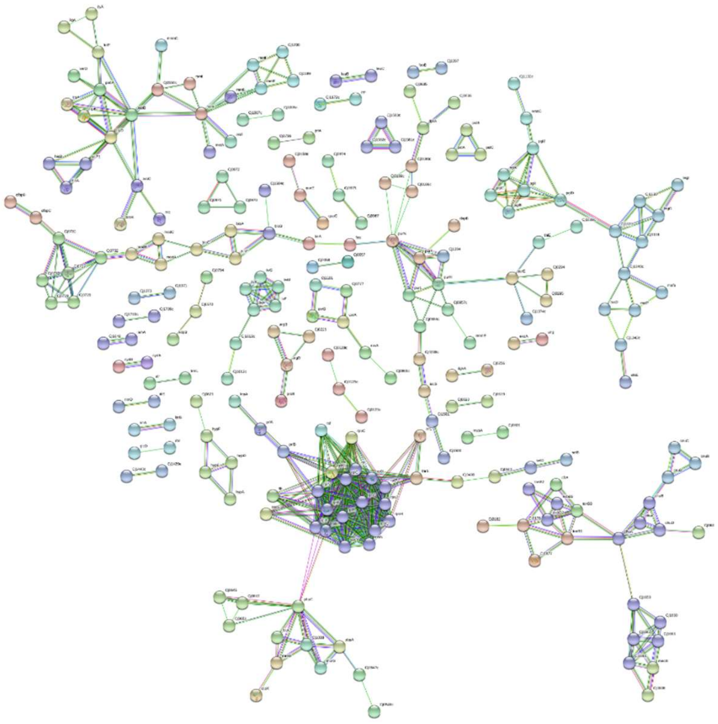

Supplementary figure S1a STRINGdb cluster analysis. DE genes with fold-change >+2, using custom assigned confidence of 0.850 and orphan nodes removed. 318 nodes, 456 edges with PPI enrichment p-value <1.0e-16

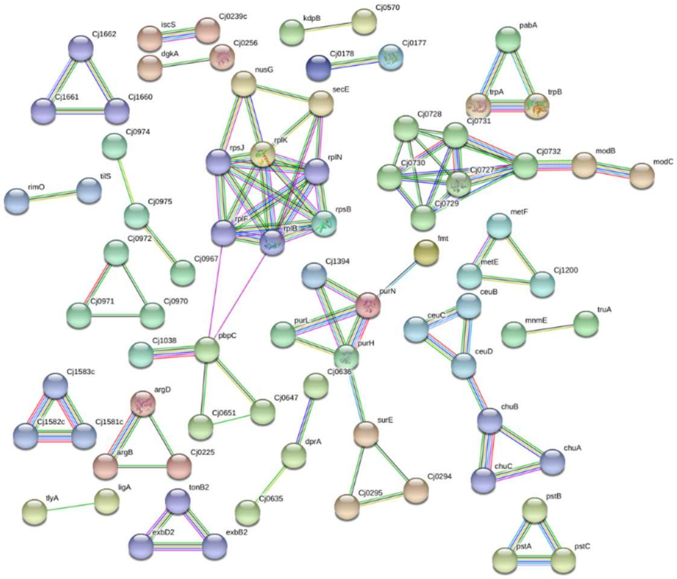

Supplementary figure S1b STRINGdb cluster analysis. DE genes with fold-change >+5 (118 genes), using custom assigned confidence of 0.850 and orphan nodes removed. 118 nodes, 96 edges with PPI enrichment p-value <1.0e-16

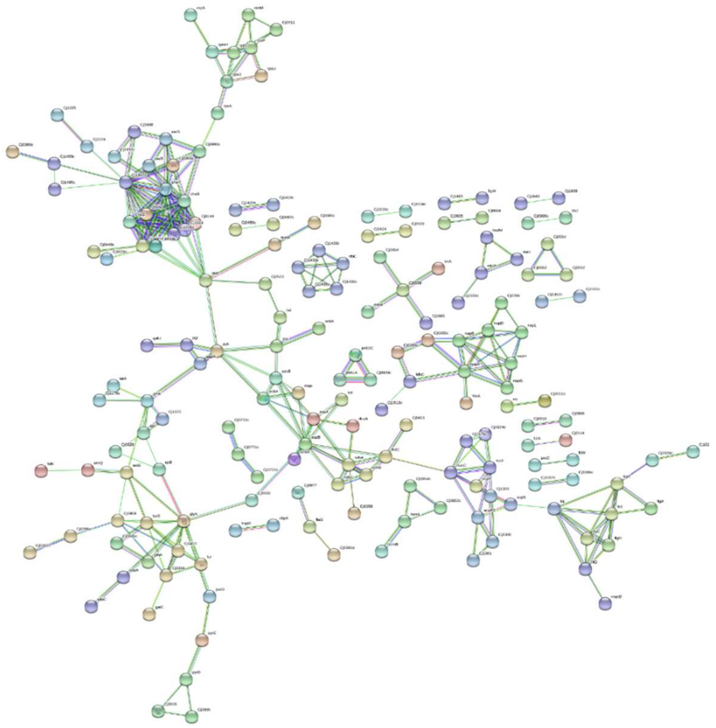

Supplementary figure S1c STRINGdb cluster analysis. DE genes with fold-change <-2, using custom assigned confidence of 0.850 and orphan nodes removed. 301 nodes, 283 edges with PPI enrichment p-value <2.2e-16

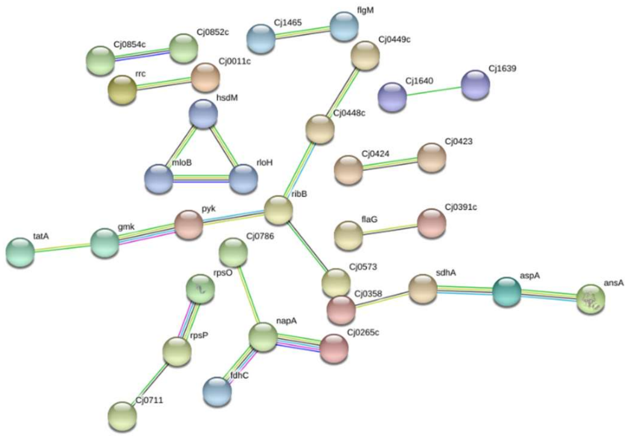

Supplementary figure S1d STRINGdb cluster analysis. DE genes with fold-change <-5 (96 genes), using custom assigned confidence of 0.850 and orphan nodes removed. 97 nodes, 23 edges with PPI enrichment p-value = 0.00043

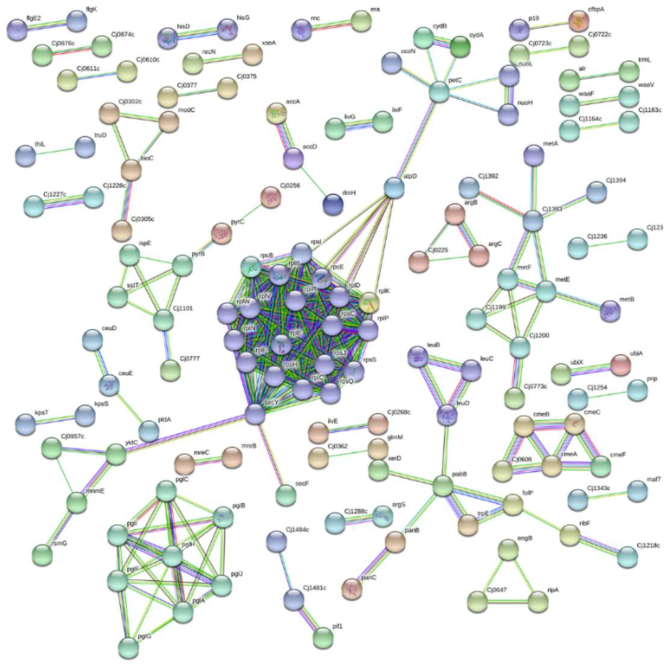

Supplementary figure S2a STRINGdb cluster analysis. DA proteins with fold-change >+1.5, using custom assigned confidence of 0.850 and orphan nodes removed. 220 nodes, 314 edges with PPI enrichment p-value <2.6e-11

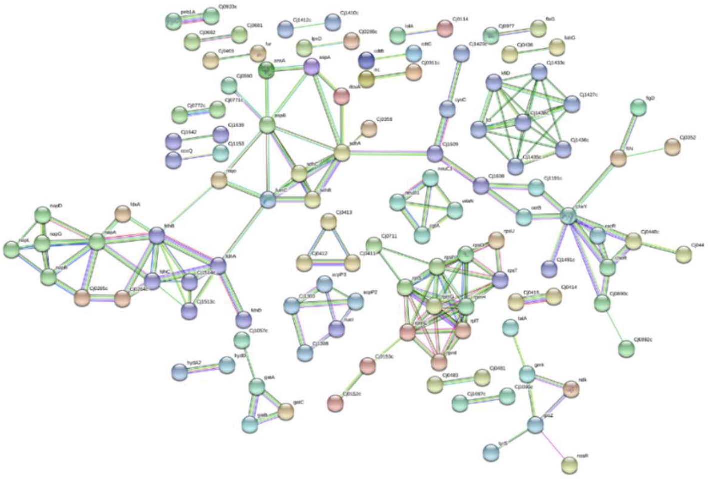

Supplementary figure S2b STRINGdb cluster analysis. DA proteins with fold-change >-1.5, using custom assigned confidence of 0.850 and orphan nodes removed. 210 nodes, 173 edges with PPI enrichment p-value <2.21e-10

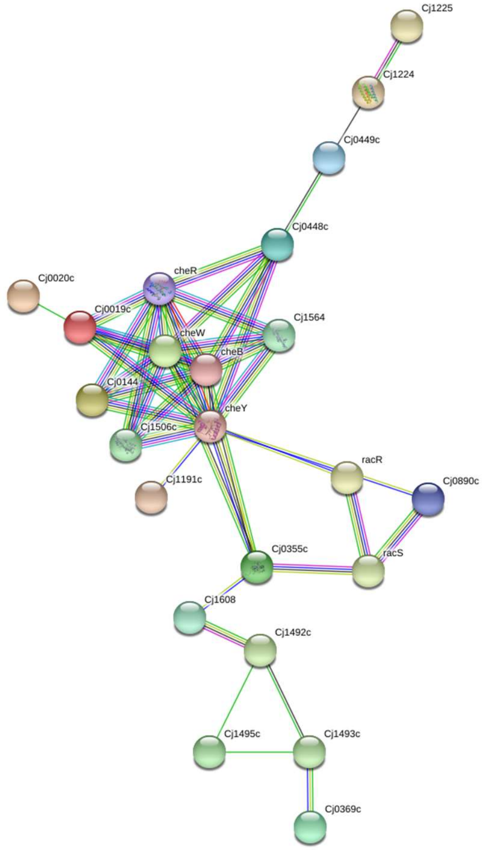

Supplementary figure S3 STRINGdb cluster of enriched chemotaxis-associated genes.

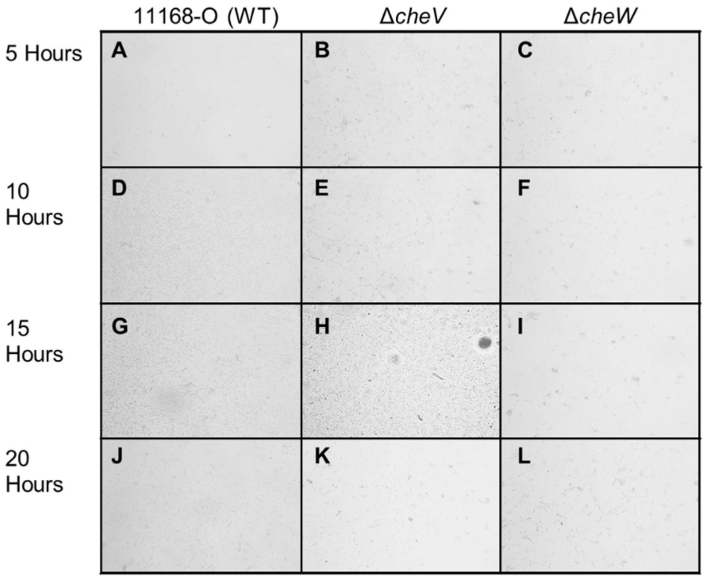

Supplementary figure S4 Time lapse microscopy. Demonstrating the tendency for aggregation of 11168-O, Δ*cheV* and Δ*cheW* isogenic mutants. Both *ΔcheV* and *ΔcheW* strains demonstrated a significantly higher tendency to cluster into microcolonies when compared to wild type 11168-O over a period of 25 hours.
